# Roles of ADP-Ribosyltransferases in Cancer

**DOI:** 10.32604/or.2026.072194

**Published:** 2026-03-23

**Authors:** Maureen Veilleux, Anh Nguyen, Charles Cao, Yihui Shi

**Affiliations:** 1 Institute of Pharmaceutical and Biological Sciences, Université Claude Bernard Lyon 1, Lyon, France; 2 California Pacific Medical Center Research Institute, Sutter Health, San Francisco, CA, USA; 3 College of Medicine, California Northstate University, Elk Grove, CA, USA; 4 Rollins School of Public Health, Emory University, Atlanta, GA, USA

**Keywords:** Cancer therapeutics, gene targets, synthetic lethality, ADP-ribosyltransferases (ARTs), poly(ADP-ribose)polymerase (PARPs)

## Abstract

ADP-ribosyltransferases (ARTs) regulate key processes in cancer, including DNA repair, transcription, immune responses, and treatment resistance. The clostridial toxin-like ADP-ribosyltransferase (ARTC) family and the diphtheria toxin-like ADP-ribosyltransferase (ARTD) family play a crucial role in genomic stability by modification of proteins either with mono(ADP-ribosyl)ation (MARylation) or poly(ADP-ribosyl)ation (PARylation). These ARTs are promising therapeutic targets and could serve as biomarkers in cancer management. This review explores the roles of these enzymes and current knowledge on specific inhibitors. A literature search was conducted in PubMed and Google Scholar to identify studies published between 1992 and 2025 on ADP-ribosyltransferases and their roles in cancer. Among ARTC family, ART1 and ART3 modulate the phosphoinositide 3-kinase (PI3K)/protein kinase B (AKT) pathway, influencing angiogenesis, tumor growth, and immune evasion via cluster of differentiation 8+ (CD8+) T-cell apoptosis. Within the ARTD family, poly(ADP-ribose)polymerase (PARP)1 and PARP2 are activated by DNA single-strand breaks and are clinically validated targets in cancers with homologous recombination deficiency, such as breast cancer susceptibility genes 1/2 (BRCA1/2)-mutated breast cancer. Their inhibition exemplifies synthetic lethality and has shown clinical efficacy. Four PARP inhibitors, olaparib, niraparib, rucaparib, are approved by the Food and Drug Administration (FDA) approved. Despite these advances, selective inhibitors for ARTs remain underexplored. Ongoing research focuses on overcoming PARP inhibitor resistance, improving biomarker-driven patient selection, and expanding therapeutic strategies that target ART-related pathways.

## 1 Introduction

ADP-ribosyltransferases (ARTs) utilize a NAD+-dependent modification to alter protein functions. The two main forms of these modification processes are mono-ADP-ribosylation (MARylation) and poly-ADP-ribosylation (PARylation). MARylation is still understudied, but there is a greater number of mono(ADP-ribosyl)transferases than poly(ADP-ribosyl)transferases. Current research points to MARylation as being capable of modifying a variety of amino acid residues, while PARylation mostly modifies serine residues. Thus, MARylation can potentially play roles in several biological processes, including DNA damage repair and inflammatory responses [1]. The function of PARylation in DNA damage repair pathways and its role with ubiquitination of PARylated proteins has been well-studied [2]. There are two major enzyme families capable of producing ADP-ribosylated proteins. The first is the clostridial toxin-like ADP-ribosyltransferase (ARTC) family, which includes four human members, two active mono-ADP-ribosyltransferases and two enzymatically inactive proteins [3]. ARTC proteins are usually extracellular and membrane-bound or secreted proteins and MARylate their substrates. The second is the diphtheria toxin-like ADP-ribosyltransferase (ARTD) family, comprising 17 proteins that function as either poly-ADP-ribosyltransferases, mono-ADP-ribosyltransferases, or inactive enzymes [3]. ARTD1-6 share the histidine-tyrosine-glutamate motif. This motif is required for facilitating the transfer of the ADP-ribose from the NAD+ cofactor for PARylation. The remainder of the ARTD family have a similar motif where the glutamate is replaced with isoleucine, leucine, threonine, valine, or tyrosine. These differences could contribute to the MARylation activity of ARTD7-17 [4]. Table 1 depicts the ARTC and ARTD proteins with the enzymatic status and ADP-ribosylation type.

**Table 1:** Mammalian ADP-Ribosyltransferase enzymes.

Family	Enzymatic Status	ADP-Ribosylation Type	Protein Name	Involvement in Cancer	Notable References
**ARTC**	Active	Mono	ARTC1/ART1	Non-small-cell lung cancer, Colorectal cancer, Glioblastoma, Cervical cancer	[9–11]
	Active	Mono	ARTC5/ART5	N/A	N/A
	Inactive		ARTC3/ART3	Breast cancer	[12,13]
	Inactive		ARTC4/ART4	N/A	N/A
**ARTD**	Active	Poly	ARTD1/PARP1	Breast cancer, Ovarian cancer, Metastatic prostate cancer, Pancreatic cancer, Small-cell lung cancer, Colorectal cancer	[14,15]
	Active	Poly	ARTD2/PARP2	Breast cancer, Ovarian Cancer, Metastatic prostate cancer	[16–18]
	Active	Mono	ARTD3/PARP3	Glioblastoma, Breast cancer, Non-small-cell lung cancer, Prostate cancer, Pancreatic cancer	[19,20]
	Active	Mono	ARTD4/PARP4	Primary thyroid and breast cancers, Lung cancer	[21–23]
	Active	Poly	ARTD5 /tankyrase1	Colon carcinoma,Breast cancer, Gastric cancer, Lung cancer	[24–26]
	Active	Poly	ARTD6 /tankyrase2	Colon carcinoma,Breast cancer, Gastric cancer, Lung cancer	[24,25,27]
	Active	Mono	ARTD7/PARP15	N/A	N/A
	Active	Mono	ARTD8/PARP14	Diffuse large B-cell lymphoma, Multiple myeloma, Hepatocellular carcinoma, Acute myeloid leukemia, Pancreatic cancer	[28–30]
	Active	Mono	ARTD10/PARP10	Oral squamous cell carcinoma, Acute myeloid leukemia, Breast and ovarian cancer	[30,31]
	Active	Mono	ARTD11/PARP11	Multiple cancers	[32,33]
	Active	Mono	ARTD12/PARP12	Breast cancer	[34,35]
	Active	Mono	ARTD14/PARP7	Lung cancer, Breast cancer	[36–38]
	Active	Mono	ARTD15/PARP16	N/A	N/A
	Active	Mono	ARTD16/PARP8	N/A	N/A
	Active	Mono	ARTD17/PARP6	Colorectal cancer, Gastric cancer	[39,40]
	Inactive		ARTD9/PARP9	Diffuse large B-cell lymphoma, Metastatic prostate cancer	[41,42]
	Inactive		ARTD13/PARP13	Liver cancer, Colon cancer, and Bladder cancer	[43]

Note: Abb: N/A: Not Applicable; ART: ADP-Ribosyltransferases; PARP: Poly(ADP-Ribose)Polymerase.

Recent studies have shown that inhibition of PARPs affects the ability of cancer cells with homologous recombination (HR) deficiencies to repair DNA [5]. Most research on poly(ADP-ribose) polymerases has focused on the functions of PARP1 and PARP2, as well as the development of their inhibitors. Studies in human cell lines have also linked PARPs to specific cancers and resistance to specific cancer treatments. For example, PARP9, PARP13, and PARP14 are linked to colorectal cancer resistance to radiotherapy [6]. Other studies have also implicated inhibition of less-studied PARPs in enhancing antitumor immunity in murine models, highlighting the importance of expanding research into these less-studied PARPs in hopes of discovering new therapeutic targets [7,8].

This review mainly explores the roles of the enzymes in the ARTD family to determine their impact on cancer while summarizing the functions of the active ARTC proteins.

## 2 Materials and Methods

This review was conducted by searching the scientific databases PubMed (https://pubmed.ncbi.nlm.nih.gov/) and Google Scholar (https://scholar.google.com/) for studies related to ADP ribosyltransferases and their roles in cancer. The initial search strings included these words: “ARTC”, “ARTD”, “ADP ribosyltransferase”, “poly(ADP-ribose)polymerase” (“PARP”). Further narrowing down of the search to specific protein names and the addition of a keyword in an “AND” Boolean operator in PubMed which were “cancer” and “inhibitor”. The two searches were used to identify relevant peer-reviewed publications published between 1992 and 2025 organized by each enzyme, with the most relevant articles represented in Table 1.

## 3 Results

### 3.1 Overview of a Related ART: The ARTC Family’s Role in Cancer Development

**Figure 1:**
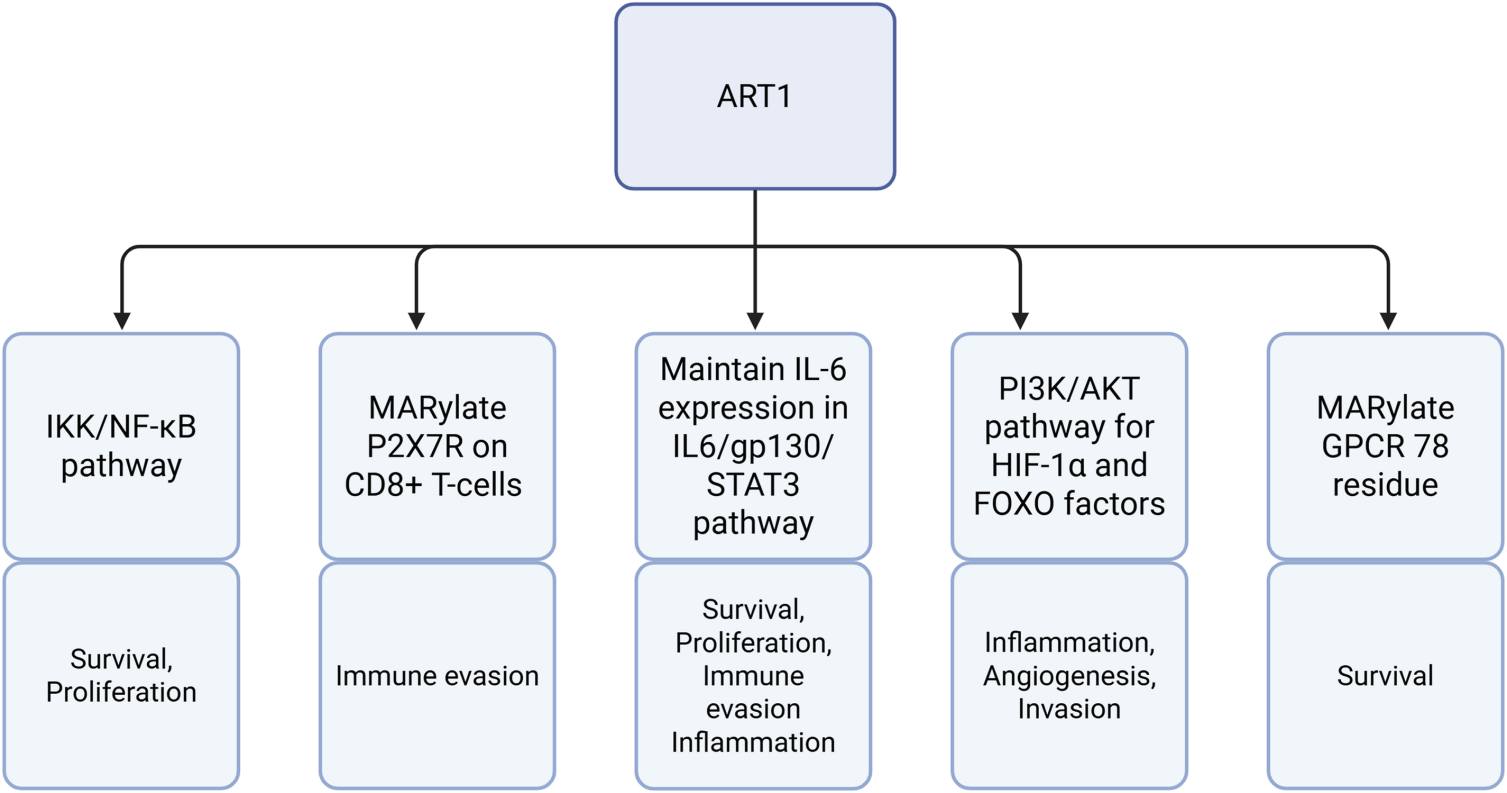
An overview of ADP-ribosyltransferase 1 (ART1)-mediated pathways and their roles in tumor biology. Abb: IKK: Inhibitor of Kappa B Kinase Complex; NF-κB: Nuclear Factor Kappa-Light-Chain-Enhancer of Activated B Cells; P2X7R: P2X7 Receptor; CD8+: Cluster of Differentiation 8+; IL-6: Interleukin-6; STAT3: Signal Transducer and Activator of Transcription 3; PI3K: Phosphoinositide-3-Kinase; AKT: Protein Kinase B; HIF-1α: Hypoxia-Inducible Factor 1-Alpha; FOXO: Forkhead Box Transcription Factor; MARylate: Mono-ADP-Ribosylate; GPCR: G Protein-Coupled Receptor.

The ADP-ribosyltransferase 1, named “ART1/ARTC1,” is a protein generally expressed at low concentration in healthy tissue. ART1 is a glycosylphosphatidylinositol-anchored enzyme that can mono-ADP-ribosylate extracellular proteins within its microenvironment, which has many roles in modulating pathways in tumor cells, as shown in Fig. 1 [44]. One of its main targets is the P2X7 receptor (P2X7R), an ATP-gated cation channel that activates inflammatory pathways. ART1 captures extracellular NAD+ to catalyze ADP-ribose transfer to P2X7R, which leads to over-activation of this receptor, triggering an uncontrolled influx of calcium that leads to externalization of phosphatidyl serine and subsequent NAD-induced cell death [45]. This NAD-induced cell death is a tumor cell evasion mechanism from the CD8+ T-cell-mediated immune response. ART1 overexpression induces a reduction in P2X7R+ CD8+ T cell infiltration in non-small cell lung cancer (NSCLC) [9]. This was underlined by ART1 knockdown in murine models, resulting in reduced tumor growth. ART1 can be a potent therapeutic target as it is an extracellular protein [9].

**Figure 2:**
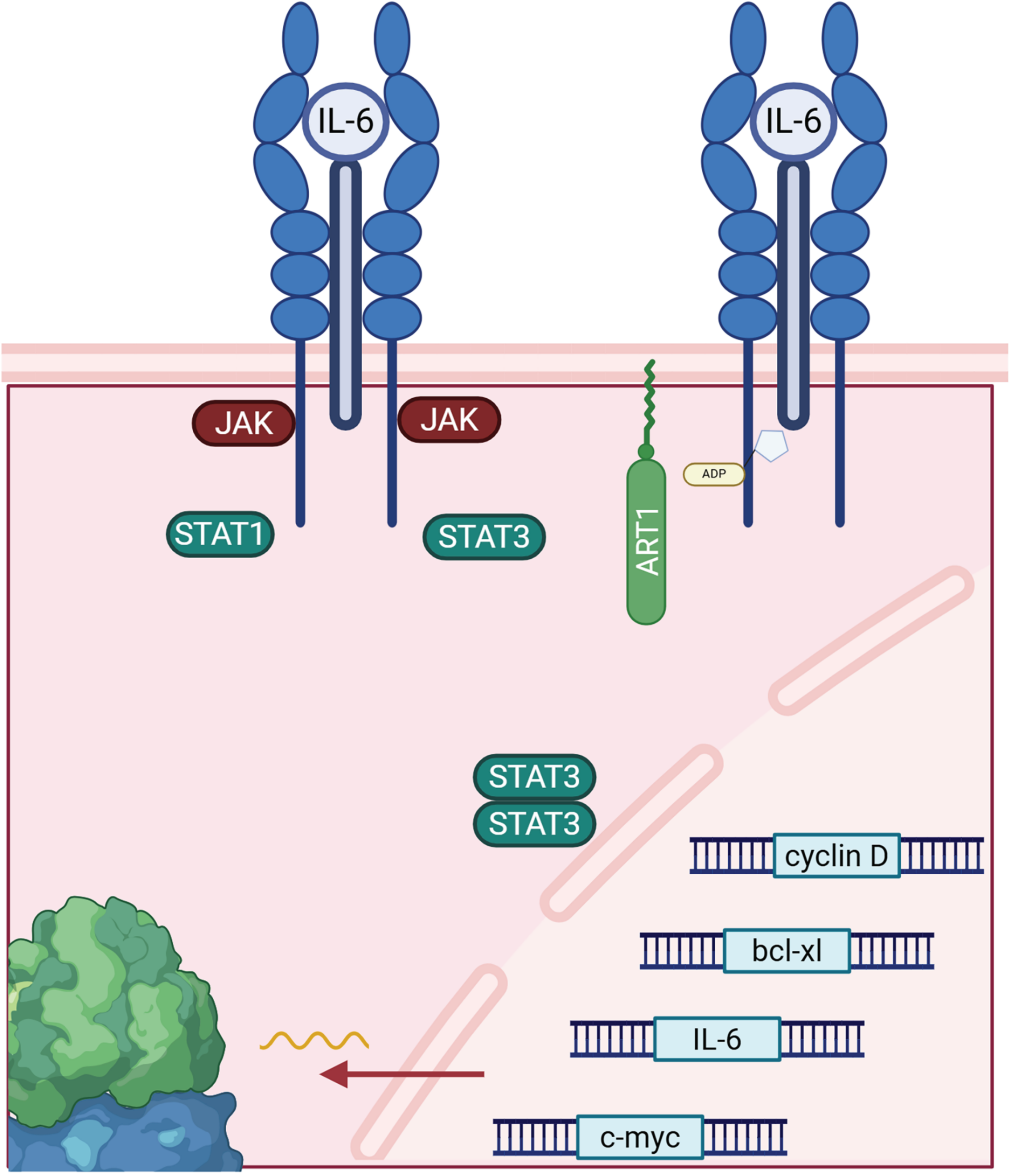
ADP-ribosyltransferase 1 (ART1) affects the interleukin-6 (IL-6)/glycoprotein 130 (gp130)/signal transducer and activator of transcription 3 (STAT3), producing more IL-6 cytokines through upregulation of gene expression from IL-6 genes and other cell survival and proliferation genes via STAT3 dimers. Abb: JAK: Janus Kinase; STAT1: Signal Transducer and Activator of Transcription 1.

**Figure 3:**
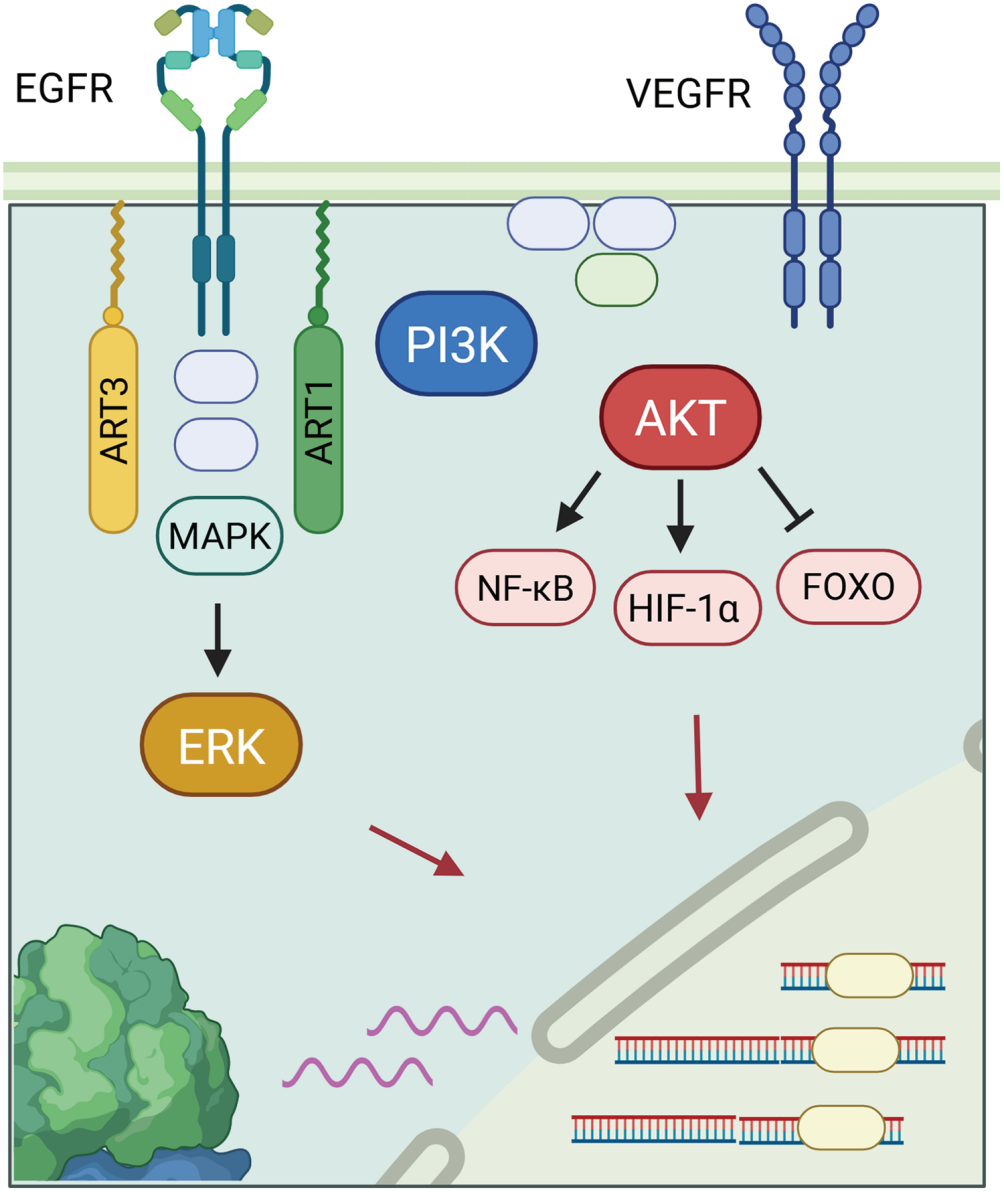
ADP-ribosyltransferase 1 (ART1) regulates the phosphoinositide-3-kinase (PI3K)/Protein Kinase B (AKT) pathway with many downstream effects on transcription factors, which include downregulating forkhead box transcription (FOXO)and its tumor suppression effects, upregulating hypoxia-inducible factor 1-alpha (HIF-1α), promoting angiogenesis through increasing transcription of human fibroblast growth factor (bFGF) and vascular endothelial growth factor (VEGF) genes, and upregulating a modified nuclear factor kappa-light-chain-enhancer of activated B cells (NF-κB) pathway for increased cell proliferation and reduced apoptosis rates [55,56]. ART3 also affects transcription of genes in cell growth and division through a closely-related mitogen—activated protein kinases (MAPK)/extracellular regulated kinase (ERK) pathway, and via Protein Kinase B (AKT).

However, according to Lin et al., ART1 seems to play a multifunctional role and can be involved in other types of cancer. For example, inhibition of ART1 reduced the expression of interleukin-6 (IL-6), a factor promoting cell proliferation in colorectal cancer [46]. Fig. 2 depicts the IL-6 cytokine generation in a positive feedback loop involving IL-6, glycoprotein 130 (gp130), signal transducer and activator of transcription 3 (STAT3). IL-6 is involved in inflammatory pathways and suppresses anti-tumor immune responses [47]. IL-6 inhibition via ART1 inhibited the STAT3 pathway, which decreases the protein expression levels of cell survival and proliferation genes. In an induced colitis murine model, ART1 knockdown inhibited IL-6/gp130/STAT3 signaling pathway and significantly suppressed tumor growth [10]. Moreover, as illustrated in Fig. 3, ART1 promotes hypoxia-inducible factor 1-alpha (HIF-1α) expression via the phosphoinositide-3-kinase (PI3K)/Protein Kinase B (AKT) pathway, thereby facilitating cell invasion and metastasis progression in colorectal cancer. HIF-1α, and therefore ART1, plays a central role in angiogenesis through over-expression of vascular endothelial growth factor (VEGF) and basic fibroblast growth factor (bFGF) [11,48]. Regulation of the PI3K/AKT pathway by ART1 was also demonstrated by a further study of colorectal cancer [49]. Complementary expression of forkhead box transcription (FOXO) factors is involved in cell proliferation and invasion. A modification of the nuclear factor kappa-light-chain-enhancer of activated B cells (NF-κB) pathway has also been highlighted, reducing apoptosis and inducing cell proliferation [50]. ART1’s role is also known in cervical cancer, where this enzyme modifies the mono-ADP ribosylation of G protein-coupled receptor 78 arginine residues in cervical cells [51]. This triggers stress in the endoplasmic reticulum, generating the Unfolded Protein Response [52]. This response enables tumor cells to adapt to their environment and survive [53]. Additionally, overexpression of ART1, also found in glioblastoma, is thought to be a marker of poor prognosis [54].

ART3, ecto-ADP-ribosyltransferase 3, catalyzes an enzymatic reaction by adding or removing an ADP ribose on an arginine of a target protein. Fig. 2 demonstrates the signaling pathways behind ART3’s role in cancer. However, its role at the molecular level still remains poorly understood. According to Tan et al. ART3 appears to be over-expressed in triple-negative breast cancers (TNBC) and basal-like cancers. TNBC is one of the most aggressive breast cancers, and the presence of ART3 is a poor prognostic factor given its association with short survival. This is likely due to increased cancer cell proliferation and decreased apoptosis. Regulation of these pathways is mediated upstream by ART3 via overexpression of the mitogen—activated protein kinases (MAPK), extracellular regulated kinase (ERK), and PI3K/AKT. ERK is involved in cell growth and division, while the PI3K/AKT pathway is involved in migration, angiogenesis, cell survival, proliferation, and tumor transformation. These pathways are thus well-suited to the development of particularly aggressive cancers. ART3 remains a highly promising therapeutic target and a potential biomarker in TNBC [12]. Indeed, according to Lindgren et al., inhibition of ART3 blocks DNA repair and increases DNA damage, particularly double-strand breaks (DSBs). Thus, the development of an ART3 inhibitor coupled with a PARP inhibitor could prove to be a very promising therapeutic avenue for overcoming resistance to PARP inhibitors, of which will be discussed in the next sections [13].

### 3.2 The PARP1-4’s Roles in Cancer Development

**Figure 4:**
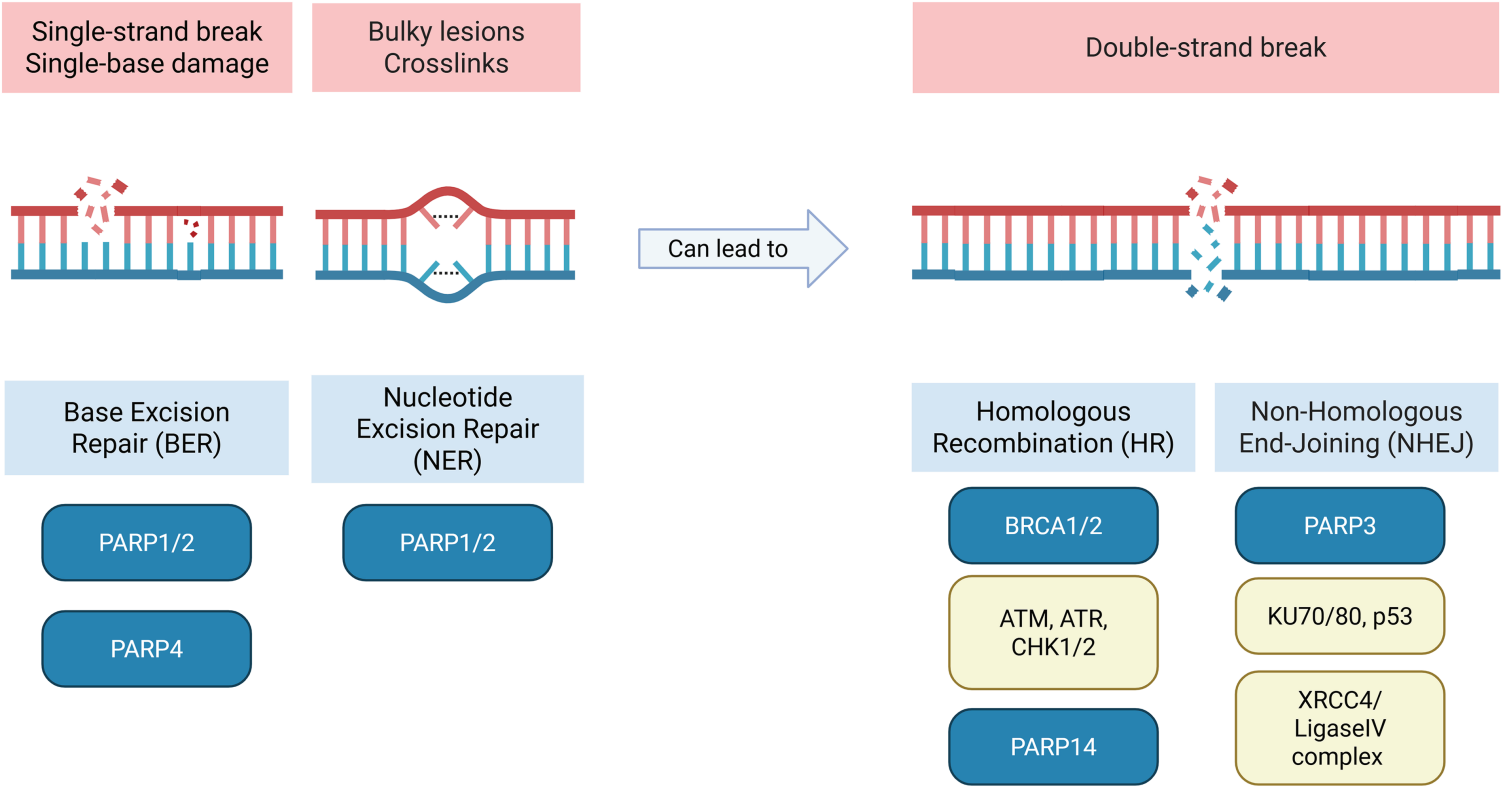
Poly(ADP-ribose)polymerase (PARP) family members in DNA repair. Schematic showing the involvement of PARP1, PARP2, PARP3, PARP4 and PARP14 in DNA repair and their associated proteins. PARP1, PARP2, PARP4 are associated with single-strand breaks (SSBs) while PARP3 and PARP14 are associated with double-strand breaks (DSBs). Abb: PARP: Poly(ADP-Ribose)Polymerase; BRCA: Breast Cancer Susceptibility Gene; ATM: Ataxia-Telangiectasia Mutated; ATR: Protein Kinase B; CHK1/2: Checkpoint Kinase 1; XRCC4: X-Ray Repair Cross-Complementing Protein 4.

PARP1 and PARP2 are molecular sensors of the DNA breaks. They are well known in oncology, not as cancer inducers but as therapeutic targets in certain types of cancer. These molecules are activated by DNA single-strand breaks (SSBs) and play roles in the cellular response to genotoxic and oxidative stress. PARP1 and PARP2 use NAD+ as a substrate to covalently conjugate ADP-ribose to themselves and other proteins to promote chromatin relaxation and recruit additional DNA repair factors of the Base Excision Repair (BER) and Nucleotide Excision Repair (NER) mechanisms, which are shown in Fig. 4 [57–59]. PARP inhibitors are frequently used in patients with homologous recombination deficiency (HRD), which is frequently linked to BRCA1/2 mutations in breast and ovarian cancers. Inhibition of PARP1/2 leads to the non-repair of an SSB, which ultimately becomes a DSB [14,60]. These DSBs are normally repaired by the HR pathway, a mechanism in which BRCA1/2 proteins are involved. However, in patients with the BRCA1/2 mutation, this system is non-functional, resulting in cell death, demonstrating synthetic lethality. Nevertheless, the application of PARP1 inhibitors has been extended to other types of cancer, such as prostate cancer, pancreatic cancer, and small-cell lung carcinoma (SCLC), irrespective of BRCA status. Indeed, alterations in DNA repair genes, which include partner and localizer of BRCA2 (PALB2), ataxia-telangiectasia mutated (ATM), BRCA1, and Fanconi anemia complementation group A (FANCA), are common in metastatic prostate cancer (mPrC). Matéo et al.’s genomic analyses show that 8% to 12% of all mPrC have BRCA mutations, and in total, up to 20% to 25% harbor HR mutations [such as partner and localizer of BRCA2 (PALB2), ataxia-telangiectasia mutated (ATM), BRCA1, Fanconi anemia complementation group A (FANCA), etc.] [61,62]. This is particularly encouraging in highly aggressive cancers such as pancreatic cancer, where a subset of patients with HR mutations (BRCA2 and PALB2) were found to respond to Veliparib or Rucaparib [63]. Furthermore, in SCLC without inactivation of any of the HR-related genes, Pilié et al. report that high levels of the PARP1 protein were detected in DNA damage responses (DDR) [15]. DDRs are a mechanism of resistance to certain treatments. SCLC is also frequently known for its loss of tumor suppressors TP53 and RB1, and its activation of Myc, an oncogene. Loss of RB1 prevents inhibition of E2F, and this transcription factor can increase transcription of PARP1, which is itself involved in DDR [64]. Inhibition of PARP1 would thus reduce DDR and inhibit cancer progression [15].

PARP2 was identified after research into the phenomenon of persistence of PARP activity despite knockout of PARP1. While closely related to PARP1, PARP2 has a distinct function and structure from PARP1, lacking zinc finger domains and BRCT motifs [16]. PARP2 also has greater recognition specificity for DNA SSBs than PARP1. PARP2 is less abundant than PARP1 when responding to DNA strand breaks, and it is kinetically slower to arrive at DNA damage sites but persists longer [17]. In addition to its role in DNA repair, it is also activated during replicative stress and plays an important role in carcinogenesis. It acts in cooperation with PARP1 to identify stalled replication forks and allow them to restart, promoting replication and tumor progression [65]. PARP2’s tissue-specific roles have been discovered in replication stress responses. PARP2 is involved in proliferative signaling, and its overexpression can repress the Smad3/4 promoter and consequently the transforming growth factor-beta (TGF-β) pathway, an antitumor pathway at the onset of tumorigenesis. Ali et al. highlight the great potential of PARP2 inhibition, which could be used to regulate several pathways enabling tumorigenesis [66]. PARP2 also promotes tumor glycolytic metabolism and is known to inhibit Sirtuin 1 (SIRT1) [18]. By modulating SIRT1, it negatively regulates the Notch pathway, which controls blood vessel growth, thus contributing to angiogenesis.

PARP3 was first identified for its DNA DSB repair functions [19]. It is an enzyme that mono-ADP-ribosylates Tankyrase 1 (PARP5a) and the mitotic factor Nuclear Mitotic Apparatus (NuMA), both involved in microtubule dynamics and telomere integrity to regulate mitotic progression [67]. Indeed, PARP3 inhibition in glioblastoma sensitizes cells to microtubule-destabilizing agents [68]. PARP3 also catalyzes ADP ribosylation of effector proteins such as histones, chromatin remodelers, and DNA repair factors (Ku70/80, p53). Illustrated in Fig. 4, PARP3 interacts with DNA repair protein Ku80 during DNA repair and helps to shunt repair to the non-homologous end joining (NHEJ) mechanism. PARP3 participates in the NHEJ DNA repair pathway by accumulating aprataxin polynucleotide kinase-like factor (APLF), a histone chaperone protein, at damaged sites [69,70]. With PARP3 depletion in human osteosarcoma cells, APLF double-strand repair recruitment kinetics were moderately reduced. Fenton et al. state that inhibition of PARP1/2 did not affect the later accumulation of phosphorylated APLF at DNA damage sites, suggesting that APLF involvement in SSBs is mediated by PARP1/2, but later DSBs occurring from SSBs involve PARP3. In breast cancer, PARP3 may be involved in epithelial-mesenchymal transition (EMT), a critical step in cancer cell metastasis. PARP3 maintains a stem cell-like phenotype through the maintenance of octamer-binding transcription factor 4 (OCT4) and sex determining region Y-box 2 (SOX2) factors [20]. Overexpression of PARP3 may be upregulated by the accumulation of ROS due to the TGF-ß pathway. Furthermore, other studies revealed that PARP3 is involved in the mammalian target of rapamycin complex 2 (mTORC2) pathway involved in cell growth and proliferation, particularly in BRCA1-mutated TNBC. This is consistent with previous findings, since mTORC2 controls TGF-ß-mediated EMT [71,72]. Inhibition of PARP3 and consequently of mTORC2, together with the presence of the BRCA1 mutation, is sufficient to induce cell death by synthetic lethality [67]. Moreover, the appearance of ROS triggered by TGF-ß to induce EMT leads to PARP3 overexpression in cisplatin-resistant NSCLC [73]. Cisplatin resistance was studied by Varol et al. and appears to be countered via synthetic lethality by combining PARP3 silencing, G protein-coupled receptor inhibition, and platelet-derived growth factor (PDGF) signaling pathways [74]. Targeting PARP3 could thus prove highly effective in several areas, including cell growth, mitotic progression, metastatic progression, and DNA repair.

PARP4 is a poly ADP ribosyltransferase whose role remains poorly understood. Some studies demonstrate PARP4’s tumor suppressor functions, but also the opposite effect of its involvement in BER that confers resistance to chemotherapy which is shown in Fig. 4 [75]. Raval-Fernandes et al. demonstrated in murine models that mice with PARP4 knockdown had a significantly higher colon tumor incidence and multiplicity and shorter colon tumor latency than wild-type mice. However, these results were not demonstrated in other cancer types, like lung tumors [76]. Importantly, however, this PARP4 enzyme appears to play a role in DNA repair and transcriptional regulation. PARP4 is part of the cytoplasmic ribonucleoprotein complex, also known as vault, a complex particularly implicated in multidrug resistance and tumor cell insensitivity to anticancer drugs [77–79]. The lung resistance protein (LRP) is the human major vault protein (MVP) which is the structural protein in the vault complex, but has varying expression levels depending on cancer type. This varying frequency of LRP could explain the varied results of studies on PARP4’s function as a tumor suppressor. Contrary to previous studies demonstrating PARP4’s role as a tumor suppressor, Sung et al. found that PARP4 expression increases after epigenetic modification in cisplatin-resistant ovarian cancer patients [80]. Proposed to be a vault-independent function, PARP4 is also involved in the development of lung adenocarcinoma. It interacts with heterogeneous nuclear ribonucleoprotein M (hnRNPM), a protein that regulates and prevents errors in the process of mRNA splicing. Alteration or disruption of either of these two partners would lead to splicing dysregulation, favoring tumorigenesis [21]. Finally, PARP4 and its pseudogene PARP4P2 can be genes involved in primary thyroid and breast cancers, but studies assessing PARP4 as a breast cancer susceptibility gene demonstrate mixed results [22,23,81].

### 3.3 Tankyrases

Tankyrases 1/2 (PARP5a/5b) interact with axis inhibition protein 2 (AXIN2) in the canonical Wnt/β-catenin pathway, one of the main pathways involved in cancer. This pathway maintains the stem cell phenotype and controls the cell cycle. β-catenin forms a destruction complex with APC, AXIN2, GSK3β, and CK1α and is subsequently degraded. Tankyrases poly-ADP-ribosylate and destroy AXIN2, preventing β-catenin degradation. From a therapeutic point of view, Waaler et al. demonstrated that inhibition of the PARP domain of tankyrases 1/2 stabilized AXIN2, thereby greatly reducing Wnt pathway signaling in colon carcinoma [24]. In a study by Huang et al., the mechanism behind tankyrase-regulated destruction of AXIN2 is explored. A small molecular inhibitor of the Wnt/β-catenin pathway was identified that blocked both tankyrase isoforms, which increased protein levels of AXIN2. Both tankyrase 1 and 2 interact with a highly conserved domain of AXIN2 and stimulate its degradation through PARylation-mediated ubiquitination. Interestingly, inhibition of only one isoform of tankyrase did not increase AXIN2 protein levels [25]. Tankyrase 1 is also involved in other pathways, and its therapeutic use is not limited to the Wnt pathway. For example, it regulates GLUT4 transport in glucose metabolism and maintains telomeres through interaction with telomeric repeat binding factor 1 (TRF1). TRF1 is a DNA-binding factor regulating telomere homeostasis. Tankyrase prevents TRF1 binding to telomeres via its ADP ribosylation. It is a positive regulator of telomere elongation, enabling the cell to acquire chromosome stabilization and immortalization. Its overexpression has been found in breast cancer [82]. In BRCA1-mutated breast cancer, tankyrase 1 inhibition induced cell death, similar to PARP inhibitors [83]. Tankyrase 1 is also upregulated in gastric cancer and is associated with tumor histology differentiation and tumor stage, making it a potential biomarker in gastric cancer [26]. *In vitro* studies by Wang et al. demonstrated that tankyrase 2 mRNA and protein expression were significantly higher in highly malignant lung adenocarcinoma tissue. Silencing of tankyrase 2 promoted apoptosis rates and reduced migration rates, highlighting a potential therapeutic target for lung cancer [27].

### 3.4 Other PARPs

PARP14 is involved in regulating IL-4 levels. STAT6 binds to PARP14 and promotes its catalytic activity, leading PARP14 to mono-ADP-rybosylate of histone deacetylases HDAC2 and HDAC3. This eventually leads to transcription upregulation of IL-4, a cytokine that can promote tumor progression, and the EP4 receptor, which enables cell proliferation in colorectal cancer cells [84]. Furthermore, since STAT6 is involved in B cell survival, PARP14 will also be involved in B cell cancers such as diffuse large B-cell lymphoma and multiple myeloma in promoting cell survival [28,85]. In the case of the latter, PARP14 inhibits JNK1, a mitogen-activated protein kinase, by binding to it. In hepatocellular carcinoma, PARP14 promotes the Warburg effect, an aerobic glycolysis frequently found in cancer cells [29,30]. PARP14 is synonymous with poor prognostic value in acral lentiginous melanoma and pancreatic cancer and is thought to upregulate the NF-κB/HIF-1α pathway, promoting angiogenesis. PARP14 is also a part of the HR pathway, which is shown in Fig. 4. In Saleh et al., depleting PARP4 and DTX3L PARP14 levels reduced cell survival and proliferation in HNSCC cell lines and HeLa cells, but specific PARP14 inhibitors did not suppress tumor cell survival mechanisms [86].

PARP10 is a protein that can be both a tumor suppressor and an oncogene. It is heavily involved in DNA repair and interacts with proliferating cell nuclear antigen to promote replication. It prevents replication fork stalls from their DNA strand following attack by fork-stalling agents (UV, HU). PARP10 mono-ADP ribosylates Aurora A, activating this kinase to enable it to join centrosomes and facilitate the successful execution of the G2/M phase. In carcinogenesis, its overexpression restarts replication forks and promotes cell proliferation. It also interacts with c-myc. According to The Cancer Genome Atlas database, PARP10 is overexpressed in breast and ovarian cancers. In oral squamous cell carcinoma, it is upregulated and acts as an oncogene, associated with EMT, regulating PI3K/AKT pathways. Its expression decreases E-cadherin levels and pushes towards a mesenchymal state with strong invasive capacities, leading to poor prognostic factor [87]. According to Wang et al., PARP10 is highly expressed in acute myeloid leukemia (AML), and knockout of this gene altered AML cell proliferation *in vitro*, demonstrating that PARP10 could be a potential new therapeutic target in AML [31].

**Figure 5:**
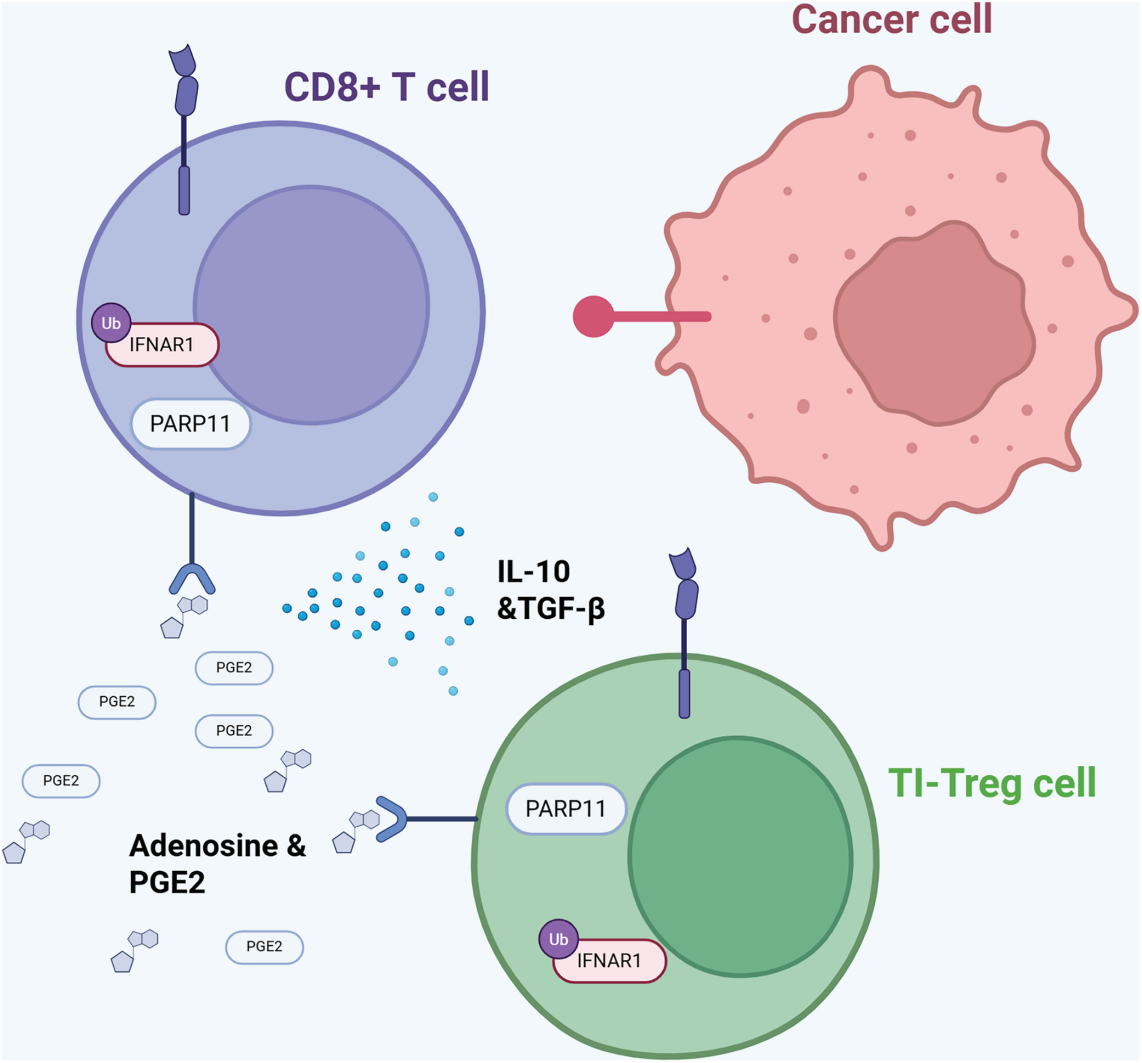
PARP11-mediated immune evasion mechanism. Diagram illustrating how PARP11 expression in tumor-infiltrating regulatory T (TI-Treg) cells downregulates INFAR1 expression in T cells, thereby promoting immune evasion [32,88]. Abb: PARP11: Poly(ADP-Ribose)Polymerase 11; CD8+: Cluster of Differentiation 8+; IL-10: Interleukin-10; TGF-β: Transforming Growth Factor Beta; PGE2: Prostaglandin E2; TI-Treg: Tumor-Infiltrating-T Regulatory Cell.

PARP11 expression is upregulated in many types of cancer, leading to tumor T regulatory cells infiltration and poor response to immune checkpoint inhibitors. Fig. 5 illustrates a pathway behind tumor cells’ immune evasion properties. The release of factors such as adenosine or prostaglandin E2 increases PARP11 expression levels within tumor-infiltrating T regulatory cells (TI-Tregs). High expression of this enzyme is linked to increased expression of β-TrCP which activates the NF-κB pathway through degradation of its inhibitor IκBα and inhibits the WNT pathway through degradation of its regulator β-catenin. Modulation of these two pathways upregulates the immunosuppressive effects of TI-Tregs through reducing inflammation that destabilizes and inactivates these TI-Tregs. Inhibition of PARP11 in this study reduced tumor Treg infiltration, secretion of immunosuppressive cytokines (IL-10, TGF-β), and improved immune checkpoint inhibitor efficacy [32]. PARP11’s immunosuppressive functions are further elucidated in Zhang et al. Type I interferon (IFN1) receptor IFNAR1 is important for CD8+ cytotoxic T lymphocytes (CTL) tumoricidal activities [33]. However, PARP11 stabilizes β-TrCP, a subunit in the E3 ubiquitin ligase complex, which facilitates the IFNAR1 ubiquitination, endocytosis, and degradation process. Even partial loss of IFNAR1 on the CTL cells contributes to the immunosuppressive effects of Tregs and the adenosine they produce [88]. Zhang et al. suggest that adenosine stimulates PARP11’s expression and catalytic activity. In this study, cells that had inhibited or downregulated PARP11 displayed some anti-tumor activity, demonstrating PARP11 inhibitors as a promising therapy for cancer [33].

**Figure 6:**
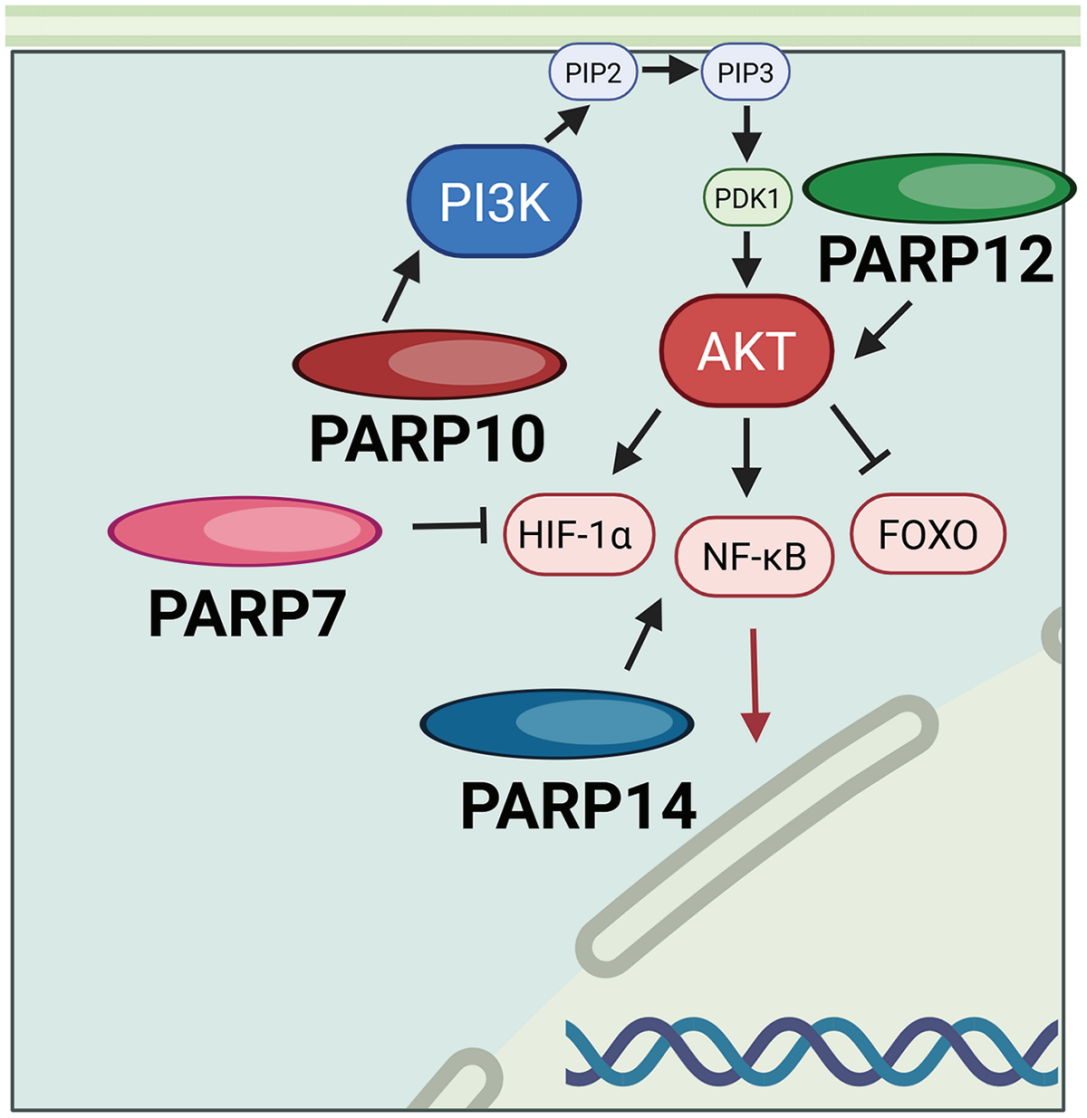
PARP10, PARP12, and PARP14 all act through the PI3K/ART or related HIF-1α, FOXO, and NF-κB pathways. Abb: PIP2: Phosphatidylinositol Phosphate 2; PIP3: Phosphatidylinositol Phosphate 3; PI3K: Phosphoinositide 3-Kinase; PARP: Poly(ADP-Ribose)Polymerase; PDK1: Phosphoinositide-Dependent Kinase-1; AKT: Protein Kinase B; HIF-1α: Hypoxia-Inducible Factor 1-Alpha; FOXO: Forkhead Box Transcription Factor; NF-κB: Nuclear Factor Kappa-Light-Chain-Enhancer of Activated B Cells.

PARP12’s function is largely unknown and understudied. The molecular basis of PARP12’s role in drug resistance in breast cancer is still unknown. However, silencing PARP12, a protein in the STING/IFN/STAT1 pathway, reduced breast cancer cell growth and survival *in vitro* [34] shown in Fig. 6, PARP12 is involved in the canonical PI3K/AKT/FOXO pathway, where mono-ADP-ribosylation of AKT is required for AKT activation. The absence of PARP12 leads to apoptosis of breast cancer cells. Inhibition of PARP12 led to increased DNA damage-induction and enhanced p53-AKT interaction; thus, PARP12 may be another potent therapeutic target for breast cancer [35].

PARP7 promotes tumor growth and suppresses the immune system. Gozgit et al. point out that high nucleic acid accumulation in the cytosol is synonymous with genetic instability in cancer. This will trigger Pattern Recognition Receptor signaling and a subsequent increase in type I INF. To prevent over-stimulation of the immune response, lung cancer cells will use PARP7 to suppress this response. PARP7 ADP-ribosylates TBK1 and inhibits it. TBK1 can no longer phosphorylate IRF3/7, the type I INF transcription factor [36]. Parsons et al. report that in ovarian cancer cells, PARP7 MARylates α-tubulin, promoting microtubule instability, a process that may contribute to cancer progression by facilitating more efficient microtubule depolymerization and disassembly. Thus, this increases the rate of ovarian cancer cell proliferation and migration [37]. In contrast to these studies, other studies have stated that PARP7 functions as a tumor suppressor. In a negative feedback loop, PARP7 represses the oncogenic actions of estrogen receptor alpha (Erα) in 17β-estradiol (E2) responsive breast cancer cells by MARylation. PARP7 knockout cells demonstrated increased ERα activity and cell proliferation in response to E2 [89]. Zhang et al.’s study also revealed that PARP7 recruits both HIF-1α and E3 ubiquitin ligase for ubiquitination and degradation of HIF-1α which is depicted in Fig. 6 [38]. However, PARP7 inhibitors were evaluated for anti-tumor activity and have demonstrated promising results [90].

PARP6 plays varied roles depending on the type of cancer in which it is involved. In colorectal cancer, it inhibits cell growth and is therefore a good prognostic factor. It leads to the accumulation of S-phase cells [39]. PARP6 has been shown in the literature to be tumorigenic in gastric cancer and colorectal adenocarcinoma. It promotes cell proliferation, migration, and invasion by increasing the expression of Survivin, a member of the apoptosis-inhibiting proteins that enables cell proliferation [40,91].

PARP9, also known as BAL1 or ARTD9, is involved in lymphoma, prostate cancer, and metastases. In diffuse large-cell lymphoma, tumor cells acquire resistance to doxycycline, resulting in increased levels of IFN-γ activating a PARP9 promoter. This overexpression of PARP9 is associated with modulation of inflammatory activity, contributing to tumor survival and immunosuppression within the microenvironment [41]. In metastatic prostate cancer, PARP9 is overexpressed via the interleukin-6/Janus kinase-signal transducer and activator of transcription (IL6/JAK1-STAT1) pathway in the absence of IFNγ or via the IFNγ/STAT1 pathway in the presence of this cytokine. PARP9 then acts in collaboration with deltex E3 ubiquitin ligase 3L (DTX3L) to inhibit the expression of IRF1, a tumor suppressor. Inhibition of IRF1 promotes an anti-apoptotic environment, contributing to tumor progression. In addition, PARP9 cooperates with PARP14 to support tumor cell survival and proliferation, notably by modulating pro-survival signaling pathways [42].

PARP13 is an antiviral factor and RNA-binding protein that modulates and inhibits specific RNAs. PARP13 regulates cellular responses to TNF-related apoptosis-inducing ligand (TRAIL) through RNA decay of the TRAIL receptors. Despite not being catalytically active, PARP13 can function as a recruiting factor for effector proteins that bind to specific overexpressed transcripts of TRAIL3 and TRAIL4. One of PARP13’s most regulated transcripts is TRAILR4, which encodes a decoy receptor for TRAIL. By inhibiting TRAILR4 through binding to its 3’UTR region, PARP13 downregulates TRAILR4’s sequestering of TRAILR1 and TRAILR2 pro-apoptotic receptors, thus activating apoptosis and killing cancer cells. PARP13, therefore, appears to be a promising therapeutic target, even though it is not catalytically active. Decreased PARP13 levels have been shown in human liver, colon, and bladder cancer samples. However, further studies are important to elucidate whether inhibiting or upregulating PARP13 activity is an effective and tolerable therapeutic strategy for cancer [43]. The PARPs functions are summarized in Table 2.

**Table 2:** PARP Family member functions.

PARP Family Member	Modification for Cancer Progression
PARP1	DNA repair mechanisms (NER, BER)
	Loss of tumor suppressors (e.g., p53, TP53 and RB1)
PARP2	DNA repair mechanisms (NER, BER)
	Angiogenesis (SIRT1/Notch)
PARP3	DNA repair mechanisms (NHEJ)
	Metastasis (EMT)
	Cell growth, survival, metabolism (mTORC2)
PARP4	Potential tumor suppressor
	DNA repair mechanisms (BER)
	Drug resistance (human MVP complex)
PARP5a/5b	Cell cycle control through Wnt/β-catenin (AXIN2 degradation)
	Glucose metabolism (GLUT4 transporter)
PARP6	Potential tumor suppressor (downregulate Survivin)
	Upregulate Survivin
PARP7	Potential tumor suppressor
	Immune system suppression
	Degradation of HIF-1α
PARP9	Drug resistance
	Inhibit tumor suppressor (IRF1)
PARP10	Potential tumor suppressor
	DNA repair mechanisms (forks)
	Metastasis (EMT through PI3K/Akt pathway)
PARP11	Tumor Treg infiltration
	Inhibit tumoricidal activities of CD8+ cytotoxic T lymphocytes (loss of INFAR1)
PARP12	Potential tumor suppressor
	Drug resistance
	Cell growth and survival (PI3K/Akt/FOXO)
	Inhibit tumor suppressors (p53)
PARP13	Potential tumor suppressor (TRAIL receptors)
PARP14	DNA repair mechanisms (HR)
	Angiogenesis (NF-κB/HIF-1α)
	Warburg effect, aerobic glycolysis (JNK1 inhibition)
	Regulate IL-4 levels

Note: Abb: NER: Nucleotide Excision Repair; BER: Base Excision Repair; NHEJ: Non-Homologous End Joining; EMT: Epithelial-to-Mesenchymal Transition; GLUT4: Glucose Transporter Type 4; IRF1: Interferon Regulatory Factor 1; JNK1: c-Jun N-Terminal Kinase 1; PARP: Poly(ADP-Ribose)Polymerase.

### 3.5 Current Clinical Applications and Potential Development of the Food and Drug Administration (FDA)-Approved PARP Inhibitors

Future development aims to expand indications and to optimize biomarker-driven patient selection to maximize the therapeutic potential of PARP inhibition. Four PARP inhibitors, a summary of each is shown in Table 3, including olaparib, niraparib, rucaparib, and talazoparib, are currently FDA-approved for the treatment of BRCA1/2-mutated malignancies, including ovarian, breast, prostate, and pancreatic cancers. The significant clinical trials for these four PARP inhibitors are summarized in Table 4. In ovarian cancer, olaparib, niraparib, and rucaparib are used both as maintenance therapy in platinum-sensitive disease and as treatment for recurrent or advanced cases, with phase III trials demonstrating significant progression-free survival (PFS) benefits. Olaparib phase III clinical trials include NCT01874353 (SOLO2,) NCT01844986 (SOLO1), NCT02477644 (PAOLA-1) trials [92–95]. Niraparib phase III clinical trials include NCT03522246 (ATHENA), NCT02655016 (PRIMA), and NCT03709316 (PRIME, conducted solely in China) [96–98]. Rucaparib phase III clinical trials include NCT01968213 (ARIEL3) and NCT02855944 (ARIEL4) [99–101]. Rucaparib has been more extensively investigated for its clinical efficacy and safety profile in maintenance cancer management, showcasing its promising therapeutic approach. Platinum-based induction chemotherapy followed by maintenance PARPi therapy did not improve outcomes for patients with metastatic castration-resistant prostate cancer (mCRPC) broadly selected for homologous recombination repair (HRR) deficiency. However, results were promising in the more stringently selected group with BRCA gene alterations [102]. Overall survival rates were similar between the treatment arms. Further studies comparing this approach to PARPi monotherapy are warranted. PFS benefit with rucaparib was maintained through the subsequent therapy line. These data support rucaparib as maintenance treatment for recurrent ovarian carcinoma [100].

**Table 3:** PARP inhibitor summaries.

PARP Inhibitor	Structure	Protein Target	IC50
Olaparib	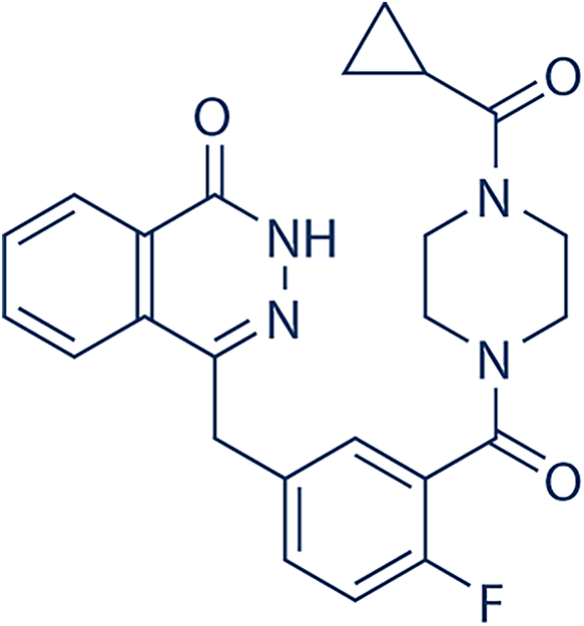	PARP1 PARP2	5 nM for BRCA1/2 deficient cancers [122] 1 nM [122]
Niraparib	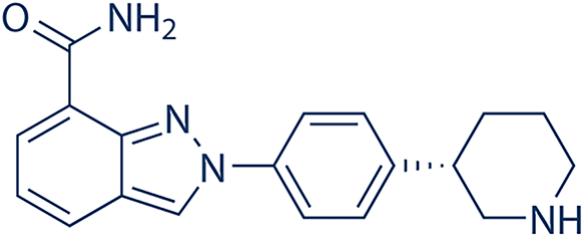	PARP1 PARP2	3.8 nM [123] 2.1 nM [123]
Rucaparib	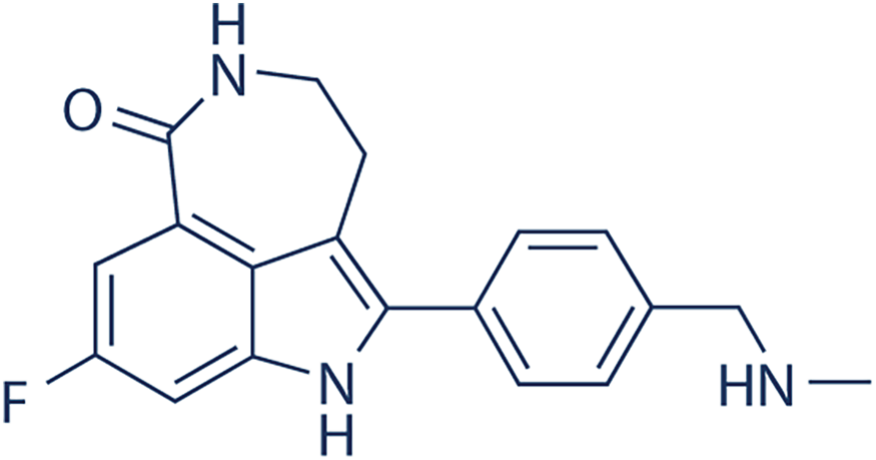	PARP1	1.4 nM (K_i_ value) [124]
Talazoparib	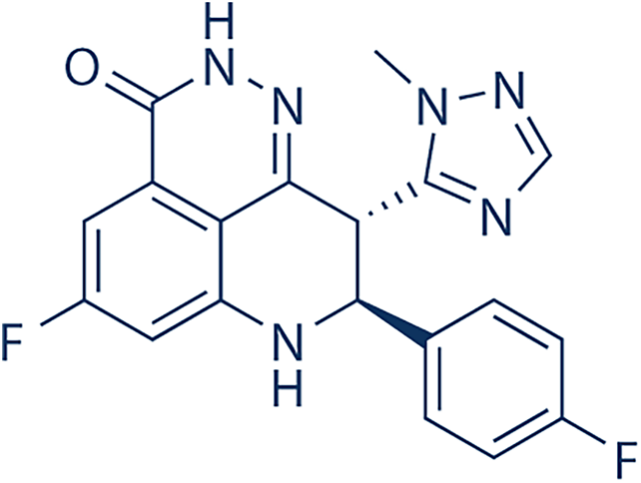	PARP1	0.57 nM [125]

Note: Abb: IC50: Half-Maximal Inhibitory Concentration. PARP1/2: Poly(ADP-Ribose)Polymerase 1/2; BRCA1/2: Breast Cancer Susceptibility Gene 1/2.

**Table 4:** Clinical application and trials.

PARP Inhibitor	Phase	Clinical Trial	Cancer	References
Olaparib	III	NCT01874353 (SOLO2)	Ovarian	[92]
Olaparib	III	NCT01844986 (SOLO1)	Ovarian	[93,94]
Olaparib	III	NCT02477644 (PAOLA-1)	Ovarian	[95]
Niraparib	III	NCT03522246 (ATHENA)	Ovarian	[96]
Niraparib	III	NCT03709316 (PRIME)	Ovarian	[97]
Niraparib	III	NCT02655016 (PRIMA)	Ovarian	[98]
Rucaparib	III	NCT01968213 (ARIEL3)	Ovarian	[99,100]
Rucaparib	III	NCT02855944 (ARIEL4)	Ovarian	[101]
Olaparib	III	NCT02000622 (OlympiAD)	Breast	[103]
Olaparib	III	NCT02032823 (OlympiA)	Breast	[104,105]
Olaparib	III	NCT03286842 (LUCY)	Breast	[126]
Talazoparib	III	NCT01945775 (EMBRACA)	Breast	[106]
Niraparib	II	NCT04240106 (LUZERN)	Breast	[127]
Olaparib	III	NCT02987543 (PROfound)	Prostate	[110]
Olaparib	III	NCT03732820 (PROpel)	Prostate	[111]
Niraparib	III	NCT03748641 (MAGNITUDE)	Prostate	[113]
Rucaparib	II	NCT02952534 (TRITON2)	Prostate	[114]
Rucaparib	III	NCT02975934 (TRITON3)	Prostate	[115]
Talazoparib	III	NCT03395197 (TALAPRO-2)	Prostate	[112]
Olaparib	III	NCT02184195 (POLO)	Pancreatic	[116]
Olaparib	I	NCT00535353	Colorectal	[119]
Olaparib	II	NCT00912743	Colorectal	[120]
Niraparib	II	NCT0332619 (OVARIO)	Ovarian	[121]

Note: Abb: PARP: Poly(ADP-Ribose)Polymerase.

In breast cancer, olaparib and talazoparib are approved for HER2-negative, germline BRCA1/2-mutated metastatic disease and, in the case of olaparib, as adjuvant maintenance in early-stage disease after chemotherapy, with improvements in PFS, invasive disease-free survival, and overall survival. The phase III clinical trials for olaparib are NCT02000622 (OlympiAD), NCT02032823 (OlympiA), and NCT03286842 (LUCY trials) [103–105]. The phase III clinical trial for talazoparib is the NCT01945775 (EMBRACA) trial [106]. A multicenter, open-label, phase II clinical trial evaluating the efficacy and safety of niraparib with aromatase inhibitors for patients with HR-positive/HER2-negative advanced breast cancer with either a germline BRCA1/2 mutation or wild-type BRCA1/2 showed that niraparib combined with an aromatase inhibitor (AI) has encouraging antitumor activity and a manageable safety profile in patients with AI-resistant HR-positive/HER2-negative advanced breast cancer with germline BRCA1/2 mutations [107]. Talazoparib was approved in 2019 for the treatment of advanced or metastatic breast cancer with BRCA gene mutation [108]. For mCRPC, olaparib is indicated in patients with BRCA1/2 or other HRR mutations following androgen receptor–directed therapy. Olaparib combined with chemotherapy enhances short- and long-term efficacy, improves immune function, and prolongs survival in advanced TNBC without increasing treatment-related toxicity, supporting its clinical utility [109]. Olaparib’s results are supported by results from the phase III clinical trial PROfound [110]. Olaparib received approval as a monotherapy following the NCT02987543 (PROfound) study and in combination with abiraterone following the NCT03732820 (PROpel) study for mCRPC. Both PROfound (HRR mutation biomarker-selected) and PROpel (biomarker unselected) patients demonstrated statistically significant longer radiographic progression-free survival (rPFS) with olaparib vs. their respective control arms in the intention-to-treat population. In both studies, the greatest clinical benefit and overall survival with olaparib was seen in patients with BRCA1 and/or BRCA2 mutations (BRCAm): PROfound HR 0.63 (95% CI 0.42–0.95); PROpel HR 0.29 (95% CI 0.14–0.56) [111].

Current investigations demonstrate a significant improvement in survival outcomes for patients with mCRPC, making talazoparib a promising intervention [108]. There is potential for synergy between talazoparib and androgen receptor pathway inhibitors, such as enzalutamide, in the treatment of prostate cancer. The phase III trial of talazoparib in prostate cancer NCT03395197 (TALAPRO-2) showed an overall survival advantage with the combination of talazoparib and enzalutamide (45.1 months) compared with placebo and enzalutamide (31.1 months) [108,112]. On August 11, 2023, the FDA approved the fixed-dose combination (FDC) of niraparib and abiraterone acetate (AA), with prednisone (P), for the treatment of adult patients with deleterious or suspected deleterious BRCA-mutated (BRCAm) mCRPC, as determined by an FDA-approved test. Substantial evidence of niraparib’s effectiveness was demonstrated by Cohort 1 of NCT03748641 (MAGNITUDE), a multi-cohort study [113]. Phase II clinical trial NCT02952534 (TRITON2) studied rucaparib and demonstrated significant clinical results in patients with mCRPC [114]. Furthermore, the Phase III clinical trial, NCT02975934 (TRITON3) demonstrated that imaging-based progression-free survival was significantly longer in patients with prostate cancer who underwent therapy with rucaparib than those who did not [115].

In pancreatic cancer, olaparib is approved for maintenance therapy in germline BRCA1/2-mutated cases without progression after first-line platinum chemotherapy in NCT02184195 (POLO), prolonging PFS compared to placebo [116]. Patient selection relies on FDA-approved companion diagnostics, such as BRACAnalysis CDx and FoundationOne CDx, to detect BRCA mutations and HRD [117,118]. In a 2016 paper on a phase I clinical trial of olaparib and irinotecan in patients with colorectal cancer, NCT00535353, in the Canadian Cancer Trials Group IND187 demonstrated a lack of antitumor efficacy [119]. Furthermore, recent phase II clinical trials of olaparib for colorectal cancer, NCT00912743, did not demonstrate significant results [120]. The phase 2, single-arm, open-label trial NCT03326193 (OVARIO) enrolled adult patients with stage IIIB-IV epithelial ovarian, fallopian tube, or primary peritoneal cancer. The median overall survival in the OVARIO trial was 61.1 months in the overall population of niraparib plus bevacizumab maintenance following first-line platinum-based chemotherapy with bevacizumab in advanced ovarian cancer [121]. Ongoing challenges include the emergence of drug resistance through mechanisms such as BRCA reversion mutations, PARP1 alterations, and restoration of HR, prompting investigation into next-generation PARP inhibitors, novel synthetic lethal partners, and combination strategies with immune checkpoint inhibitors, anti-angiogenic agents, and other DNA damage response–targeting drugs.

### 3.6 Development of Selective PARP Inhibitors

After the discovery of PARP1 inhibitors 30 years ago, benzamides and isoquinolines are early PARP inhibitors that established a core pharmacophore for future PARP inhibitors [128]. Table 5 details the current PARP inhibitor research. By the late 90s, the identification of a second PARP, termed PARP2, was reported [16]. Since PARP2 carries out the same catalysis as PARP1, uses the same co-substrate, and is highly homologous, it is not surprising that most PARP inhibitors show similar inhibition potency between both PARPs [129]. In the case of PARP1, the roles of recognition of DNA damage and repair in the base excision pathway are well established. Generation of SSBs tends to accumulate in cells treated with PARP inhibitors, but this is not the case in cells treated with PARP1 siRNA [130]. Current PARP1 inhibitors include 3-aminobenzamide, NU1025, AG14361, and olaparib [131,132]. The current PARP6 inhibitor under study is AZD2281 [133]. The function and expression of mono-ARTs differ depending on the types of cancer. PARP3 and PARP9 are overexpressed in BRCA1-associated cancer and DLBCL [134]. While PARP7 expression is not enhanced in breast, liver, and colorectal cancer, its expression favors favors tumor progression in ovarian cancer [37]. PARP14 is critical for multiple myeloma cell survival [135]. PARP1/2 inhibitors (olaparib, niraparib, and rucaparib) are used in the treatment of recurrent ovarian cancers as maintenance therapy. PARP inhibitors therapy has revolutionized the treatment of ovarian cancer, but significant challenges remain. Patients with BRCA1/2 wild-type, HR-proficient biomarker statuses experienced less benefit from PARP inhibitors, with niraparib and rucaparib demonstrating significant survival advantages [136].

**Table 5:** Overview of Poly(ADP-Ribose)Polymerase (PARP) inhibitors.

Inhibitor Name	Protein Target(s)	References
3-aminobenzamide	PARP1	[131]
NU1025	PARP1	[131]
AG14361	PARP1	[131]
Olaparib (AZD2281)	PARP1	[132]
AEP07	PARP4 tankyrase1 tankyrase2 PARP15	[139]
UD	PARP14	[36]
UD	PARP10	[36]
ITK7	PARP11 PARP12	[138]
RBN-2397	PARP7	[36,90]
UD	PARP16 PARP8	[36]
AZ0108	PARP6 PARP9 PARP13	[133]

Note: Abb: UD: Undisclosed.

The development of selective inhibitors of mono-ARTs is garnering increasing attention. Presently, selective inhibitors of MARylating PARPs are available for PARP4, 6, 7, 10, 11, 14, and 16, and only one PARP7 inhibitor, RBN-2397, is currently under phase I clinical trial (ClinicalTrials.gov identifier: NCT04053673) [36,137]. In 2018, Kirby et al. generated a PARP11 selective inhibitor, ITK7, by exploiting structural differences in the active regions of PARPs that facilitate MARylation vs. PARylation [138]. Recently, Kirby et al. developed a selective PARP4 inhibitor, AEP07, by utilizing structural bioinformatics approaches to target a unique threonine residue (Thr484) in the PARP4 nicotinamide sub-pocket [139].

## 4 Limitations

In cancers, cells with deficiencies in HRR mechanisms often become more dependent on the PARP-mediated repair mechanism to efficiently repair DSBs. As such, PARP inhibitors serve as key targeted therapies through synthetic lethality in the treatment of cancers with HRD. PARP1/2 inhibitors are now FDA-approved for several cancer types, including ovarian, breast, prostate, and pancreatic cancer. Beyond PARP1/2, other ARTDs such as PARP3 contribute to cancer progression by enhancing stem-like features and promoting therapeutic resistance, particularly in BRCA1-deficient breast cancer. Inhibiting PARP3 has shown synergy with vinorelbine in preclinical models, suggesting possible combination strategies in TNBC.

Despite progress, substantial challenges remain. For example, ART family members share significant structural homology, making selective inhibition technically difficult, especially for mono-ARTs, where few validated small molecules exist. Mono-ARTs such as ART3 and ART4 are also poorly characterized, and their context-specific roles in tumors and immune microenvironments are still unclear. Additionally, off-target effects, suboptimal pharmacokinetics, and compensatory DNA repair mechanisms may reduce therapeutic efficacy. Clinically, while PARP1/2 inhibitors have demonstrated success in BRCA-mutated cancers, clinical trials for other ART inhibitors are lacking. There is an urgent need for biomarker-driven trials to evaluate ART inhibitors in broader settings, including HR-proficient and PARP-refractory tumors. Integrating ART expression and activity profiling into patient stratification, along with rational combination strategies, such as immune checkpoint blockade or DNA-damaging agents, represents a promising avenue. Continued preclinical research and well-designed clinical studies are essential to fully realize the therapeutic potential of ART inhibitors in precision oncology.

## 5 Conclusions

In conclusion, ARTs hold promise as both therapeutic targets and diagnostic/prognostic biomarkers. Their inhibition, particularly in strategic combinations, offers a path toward overcoming resistance and personalizing treatment across diverse tumor types. Strategic integration of ART biology into precision oncology frameworks will be essential for unlocking its full clinical potential.

## Data Availability

Not applicable.
